# Astaxanthin as a neuroprotective modulator of synaptic plasticity, learning, and memory: mechanistic insights and therapeutic perspectives in neurodegenerative aging

**DOI:** 10.3389/fnagi.2025.1737001

**Published:** 2026-02-10

**Authors:** Fan Guo, Jiapei Chi

**Affiliations:** 1 College of Nursing and Health, Shanghai Zhongqiao Vocational and Technical University, Shanghai, China; 2 Publicity Department, Shanghai Zhongqiao Vocational and Technical University, Shanghai, China

**Keywords:** astaxanthin, cellular pathways, learning, neurodegenerative aging, neuroprotection

## Abstract

Astaxanthin (AST), a xanthophyll carotenoid derived from microalgae and marine organisms, has emerged as a potent neuroprotective compound with remarkable antioxidant and anti-inflammatory properties. Growing evidence indicates that AST can modulate multiple molecular and cellular pathways involved in neuronal survival, synaptic plasticity, learning, and memory, particularly in the context of neurodegenerative aging. This review provides an up-to-date and integrative overview of current evidence regarding AST’s mechanisms of action across experimental and preclinical models of neurodegenerative diseases such as Alzheimer’s, Parkinson’s, and Huntington’s diseases. We highlight its role in mitigating oxidative stress, regulating mitochondrial function, modulating neuroinflammatory signaling, and promoting neurogenesis and synaptic remodeling. Furthermore, we discuss how AST influences key molecular pathways that underlie cognitive function and plasticity. The translational potential of AST as a therapeutic and preventive agent is critically evaluated, alongside its pharmacokinetic challenges, bioavailability, and safety profile in aging populations. Finally, we identify current research gaps and propose future directions toward harnessing AST for promoting cognitive resilience and delaying the progression of neurodegenerative disorders. Collectively, the evidence supports AST as a promising candidate for maintaining neuronal health and cognitive function during brain aging.

## 1 Introduction

The prevalence of neurodegenerative diseases is growing, largely because of the rise in the elderly population in recent years (Heemels, 2016). These diseases, including multiple sclerosis (MS), Alzheimer’s disease (AD) and Parkinson’s disease (PD), are often characterized by progressive neuronal dysfunction and degeneration. They often result from a combination of genetic, environmental, and lifestyle factors, and share common pathological mechanisms, such as chronic oxidative stress, excitotoxicity, neuroinflammation, and mitochondrial dysfunction. AD, the leading cause of dementia, is characterized by amyloid-β plaques and tau tangles that disrupt neuronal function. PD involves degeneration of dopaminergic neurons in the substantia nigra, leading to motor dysfunction and non-motor symptoms such as sleep and cognitive disturbances (Armstrong, 2020). MS is an autoimmune-mediated demyelinating disorder, causing disrupted signal transmission and progressive neurological impairment. Although the symptoms of neurodegenerative disorders vary, all of them present with progressive neuronal degeneration in specific brain regions (Jellinger, 2010). Neurodegeneration is associated with dysfunctions in synapses and neural networks, as well as the buildup of structurally altered protein forms in the brain (Taoufik et al., 2018). While most neurodegenerative diseases progress without remission, treatments are typically aimed at reducing symptoms, relieving pain when necessary, and restoring mobility. The global rise in neurodegenerative diseases underscores the urgent need for effective prevention and treatment. These disorders reduce patients’ quality of life and place a heavy burden on caregivers and healthcare systems. Novel interventions targeting disease mechanisms are essential to slow or prevent progression (Wang and Michaelis, 2010).

Although the pathological features of these diseases are well established, the search for multi-target interventions has led to growing interest in astaxanthin (AST). Unlike many conventional antioxidants, AST crosses the blood–brain barrier and protects both hydrophilic and lipophilic compartments. Its unique pharmacological profile positions it as a promising candidate for neuroprotection. Compounds like AST, a powerful antioxidant, are being investigated for their ability to mitigate oxidative stress and inflammation, key factors in the advancement of neurodegenerative disorders. These agents protect neuronal health and slow down disease progression, offering a potential adjunct to other therapies (Sztretye et al., 2019).

Therefore, the purpose of this review is to explore the potential of AST as a treatment modality to decrease the progression and burden of neurodegenerative diseases. Specifically, we focus on the biochemical and molecular mechanisms through which AST influences cellular processes, including oxidative stress reduction, modulation of neuroinflammation, and protection against neuronal damage. By providing a comprehensive analysis of the available evidence, this paper seeks to offer insights into how AST could be utilized as part of future therapeutic strategies, ultimately contributing to the development of interventions that are more helpful in the treatment of these diseases. This review is distinct from prior analyses by focusing not only on AST’s molecular mechanisms but also on its delivery challenges, bioavailability, and translational relevance. By integrating preclinical findings with emerging therapeutic strategies, we aim to provide a forward-looking perspective on AST’s role in neurodegeneration.

## 2 The molecular and biochemical pathologies underlying neurodegenerative diseases

Neurodegenerative diseases refer to a range of disorders that are identified by the gradual deterioration of both function and structure of the nervous system. Conditions such as MS, AD, and PD share various molecular and biochemical irregularities that lead to neuronal dysfunction and death.

### 2.1 Misfolding and aggregation of proteins

In many neurodegenerative diseases, the accumulation of proteins that are misfolded plays a critical role in disease progression. In AD, plaques of amyloid-beta and neurofibrillary tangles of tau are key pathological hallmarks. The aggregation of these proteins impairs neuronal function by disrupting cellular balance and inducing toxicity. Likewise, in PD, the accumulation of alpha-synuclein in Lewy bodies contributes to neurodegeneration. Aggregation of these disrupts normal cellular processes, such as protein degradation, resulting in cellular stress and, ultimately, cell death (Tsoi et al., 2023). Recent evidence suggests that AST may interfere with these pathogenic processes by reducing amyloid-β aggregation and attenuating tau hyperphosphorylation in Alzheimer’s models, thereby mitigating protein-induced neuronal toxicity.

### 2.2 Mitochondrial dysfunction

Mitochondria are necessary for cellular energy, but in neurodegenerative diseases, mitochondrial dysfunction is a major factor in neuronal damage. In diseases like AD and PD, mitochondrial dysfunction leads to a decrease in the production of ATP while increasing the levels of reactive oxygen species (ROS). This results in oxidative damage to cellular components. This damage worsens neuronal dysfunction and accelerates disease progression. Additionally, impaired mitochondria disrupt cellular processes such as apoptosis and autophagy, further contributing to neuronal loss and pathology in these diseases (Muddapu et al., 2020). AST has been shown to preserve mitochondrial bioenergetics by lowering ROS production, maintaining ATP levels, and stabilizing mitochondrial membranes, which collectively support neuronal survival under oxidative stress conditions.

### 2.3 Oxidative stress

Oxidative stress is another important process that occurs in neurodegeneration. The imbalance between the production of ROS and the body’s defense against it leads to damage to lipids, proteins, and DNA in neurons. This damage disrupts cellular signaling and the function of mitochondria, eventually resulting in neuronal death. The brain, due to its high metabolic activity, is especially susceptible to oxidative stress, and its natural defense mechanisms, like antioxidant enzyme systems, are often inadequate in these diseases. This oxidative damage is particularly severe in the substantia nigra in PD, where dopamine-producing neurons are especially vulnerable to ROS (Liu et al., 2025). Through its unique hydrophilic and lipophilic structure, AST can neutralize ROS across different cellular compartments, offering superior antioxidant protection compared to classical compounds such as vitamin E or β-carotene.

### 2.4 Neuroinflammation

Neuroinflammation is another critical factor in the progression of neurodegenerative diseases. Chronic activation of astrocytes and microglia leads to the release of cytokines involved in inflammation, chemokines, and other inflammatory mediators. In AD, for example, inflammation exacerbates amyloid-beta accumulation and tau hyperphosphorylation, thereby worsening neuronal damage. In PD, neuroinflammation exerts an important effect on the degeneration of neurons responsible for the release of dopamine. Although inflammation can be a protective response, sustained activation of neuroinflammatory pathways leads to neuronal injury and causes the progression of neurodegenerative disorders (Adamu et al., 2024). AST modulates neuroinflammation by downregulating NF-κB signaling and inhibiting microglial activation, leading to reduced secretion of pro-inflammatory cytokines such as IL-1β, IL-6, and TNF-α.

### 2.5 Impaired protein degradation systems

Dysfunction in systems responsible for protein degradation, such as the ubiquitin-proteasome system (UPS) and autophagy, contributes to the development of AD and PD. The buildup of misfolded or damaged proteins, such as amyloid-β in AD and α-synuclein in PD, occurs due to the failure of these pathways, causing cellular stress, inflammation, and neurodegeneration. Reversing these impairments could present potential treatment modalities against the rapid advancement of these diseases (Cummings, 2003). Experimental studies suggest that AST may enhance autophagy and proteasome function, thereby facilitating the clearance of misfolded proteins and limiting cellular stress.

### 2.6 Genetic factors

Genetic mutations and polymorphisms can elevate the risk of developing neurodegenerative diseases. For example, mutations in the PSEN2, APP, and PSEN1 genes are associated with familial AD, while alterations in the LRRK2 gene have been correlated with familial PD. These genetic variations disrupt normal functions that occur in cells, ultimately leading to neurodegeneration (Kovacs, 2016). A thorough understanding of these molecular and biochemical mechanisms is vital for developing effective, targeted therapeutic agents that are able to potentially reverse the progression of neurodegenerative diseases. By addressing the underlying causes of these conditions at the cellular and molecular levels, we can create more precise interventions aimed at preserving neuronal function and slowing or preventing disease advancement. Although AST does not directly modify genetic mutations, its ability to counter downstream oxidative and inflammatory damage may mitigate the impact of genetic susceptibility on disease progression.

### 2.7 Astaxanthin and synaptic plasticity

Synaptic plasticity is a fundamental cellular mechanism underlying learning and memory, encompassing functional changes such as long-term potentiation (LTP) and long-term depression (LTD), as well as structural remodeling of synapses. Emerging evidence indicates that astaxanthin positively modulates several key components of synaptic plasticity.

In experimental models of cognitive impairment, astaxanthin treatment has been shown to enhance hippocampal LTP while preventing stress- or toxin-induced synaptic weakening, suggesting preservation of activity-dependent synaptic signaling. These functional effects are accompanied by structural changes, including increased dendritic spine density and improved synaptic integrity in hippocampal neurons.

At the molecular level, astaxanthin upregulates synaptic proteins involved in both pre- and postsynaptic function, such as synaptophysin, PSD-95, and SNAP-25, while promoting neurotrophic signaling through the BDNF/TrkB–CREB pathway. Collectively, these findings indicate that astaxanthin supports synaptic plasticity through coordinated functional, structural, and molecular mechanisms, providing a mechanistic basis for its reported cognitive benefits (Grimmig et al., 2019; Liu et al., 2021).

## 3 Biochemical and molecular mechanisms in AD

Alzheimer’s disease is considered a neurodegenerative disorder that is progressive and mainly affects cognition, such as disrupting memory, learning, and decision-making. The biochemical mechanisms of AD include hallmark features such as Aβ plaque accumulation, tau hyperphosphorylation, and impaired calcium signaling. These occur alongside shared neurodegenerative processes such as mitochondrial dysfunction, oxidative stress, and neuroinflammation.

As mentioned, one of the key molecular hallmarks of AD is the Aβ plaques, which form as a result of the abnormal processing of amyloid precursor protein (APP). The enzyme beta-secretase cleaves APP to produce Aβ peptides, particularly Aβ42, which aggregate into toxic oligomers that form plaques. These plaques disrupt synaptic function by impeding neurotransmission and triggering excitotoxicity, leading to cognitive decline. Aβ deposition is closely linked to the impairment of synaptic plasticity, a key mechanism underlying learning and memory. The hippocampus, critical for memory processing, is particularly vulnerable to Aβ-induced damage, leading to memory deficits commonly observed in AD (Selkoe and Hardy, 2016). Tau, a microtubule-associated protein, is another major player in AD pathology. Under normal conditions, tau stabilizes microtubules, which are essential for intracellular transport. However, in AD, tau is hyperphosphorylated, causing it to detach from microtubules and form insoluble tangles within neurons. These tau tangles disrupt axonal transport, impair cellular functions, and eventually lead to neuronal death. Tau pathology has been linked to the cognitive decline, particularly in areas such as the hippocampus as well as cortex, which are heavily involved in memory formation and executive function (Iqbal et al., 2010).

Another important factor in AD is the dysregulation of homeostasis in calcium levels. Neurons depend on precise calcium signaling for synaptic plasticity and neurotransmitter release. In AD, calcium influx becomes dysregulated, leading to elevated intracellular calcium levels. This disrupts various signaling pathways, including those involved in tau phosphorylation, function of synapses, and survival of neuron cells. The abnormal calcium signaling seen in AD further accelerates neuronal dysfunction and synaptic loss, particularly in brain regions critical for memory and learning (Zündorf and Reiser, 2011).

Collectively, these biochemical and molecular disruptions lead to significant impairments in brain function. Cognitive deficits, especially in memory and learning, are the primary clinical manifestations of AD and are directly linked to the dysfunction of the hippocampus and cortex. As the disease progresses, additional brain regions involved in higher cognitive functions are affected, leading to more widespread cognitive and behavioral impairments (Cummings et al., 2019).

## 4 Biochemical and molecular mechanisms in PD

Parkinson’s disease is a chronic neurodegenerative disorder that is also progressive, similar to AD, and mainly affects the motor system, though non-motor symptoms are also prevalent. The disease is identified by loss of neurons involved in the production of dopamine and located in the substantia nigra pars compacta. Therefore, there is a depletion of dopamine observed in the striatum. This imbalance significantly disrupts the basal ganglia circuitry, leading to the hallmark motor symptoms of PD, including tremors, bradykinesia, and rigidity. PD involves biochemical changes that worsen neuronal degeneration, including α-synuclein accumulation, dopaminergic neuron loss, and basal ganglia disruption. These occur alongside mitochondrial dysfunction, oxidative stress, and chronic inflammation. The degeneration of neurons producing dopamine in the substantia nigra leads to a depletion of dopamine within the striatum, disrupting the finely tuned excitatory and inhibitory signaling in the basal ganglia. This disruption manifests as the motor symptoms commonly associated with PD, such as slowed movement, muscular rigidity, and resting tremors. However, the impact of dopamine loss extends beyond motor circuits. Dopamine is also crucial for cognitive and emotional regulation. Consequently, patients suffering PD commonly present with symptoms unrelated to motor function, such as cognitive decline, depression, anxiety, and sleep disturbances. These symptoms significantly impair quality of life and often emerge before the onset of motor deficits (Ramesh and Arachchige, 2023). Emerging evidence suggests that other neurotransmitter systems, including glutamatergic, cholinergic, and GABAergic pathways, also play a role in PD pathology. For instance, disruptions in cholinergic signaling are linked to cognitive impairments, while alterations in GABAergic and glutamatergic transmission contribute to motor dysfunction and excitotoxicity, respectively (Obeso et al., 2017). PD is a multifaceted disorder involving a complex interplay of biochemical and molecular mechanisms that contribute to its progression. The interplay between α-synuclein pathology, neuroinflammation, dysfunction of mitochondria, oxidative stress, and altered protein degradation pathways culminates in neuronal degeneration. Understanding these mechanisms and their effects on brain function is essential for the development of novel therapeutic strategies aimed at halting or reversing the course of the disease.

## 5 Astaxanthin (AST)

Carotenoids have long been recognized for their positive effects on individuals’ health, with some acting as precursors to vitamin A, essential for various biological functions, while others, known as non-provitamin A carotenoids, offer protective benefits such as defense against photodamage (Adamu et al., 2024). Among these, AST, a xanthophyll carotenoid, has gained significant research interest since it plays regulatory roles in different mechanisms, such as oxidative stress, inflammation, and neuroprotection (Fakhri et al., 2019). Predominantly found in marine creatures (e.g., krill, microalgae, salmon, and shrimp), AST’s vibrant reddish-orange hue is attributed to its unique molecular structure. This structure allows AST to effectively neutralize ROS, safeguarding cellular components from oxidative damage (Zuluaga et al., 2018). Its conjugated double bonds stabilize free radicals and prevent lipid peroxidation, thus maintaining cellular integrity. What sets AST apart from many other antioxidants is its dual hydrophilic and lipophilic nature, enabling it to protect both the lipid and aqueous environments of cellular membranes (Rizzardi et al., 2022; Shastak and Pelletier, 2023). AST is particularly noted for its exceptional stability, allowing it to withstand oxidative conditions and offer prolonged, sustained protection. Notably, AST can cross the blood-brain barrier; thus, it is capable of directly affecting the central nervous system (Galasso et al., 2018). These combined properties have led to increasing interest in its therapeutic potential across various clinical contexts, including the health of the cardiovascular system, metabolic syndrome, gastric ulcers, cancer, and neurodegenerative diseases (Copat et al., 2025; Fassett and Coombes, 2012; Gao et al., 2025; Grimmig et al., 2017; Kamath et al., 2008).

Astaxanthin is recognized as a dietary supplement and is readily accessible, with no significant adverse effects reported to date, making it both a safe and cost-effective option for further investigation (Fassett and Coombes, 2011; Yoshida et al., 2010). AST (3,3′-dihydroxy-β,β’-carotene-4,4′-dione) is a xanthophyll carotenoid composed of two terminal β-ionone rings linked by a conjugated polyene chain, with a molecular formula of C40H52O4 and 13 conjugated double bonds that contribute to its distinctive red-orange color and antioxidant properties. The molecule has two asymmetric carbons at the 3 and 3′ positions on the β-ionone rings, giving rise to three optical stereoisomers: (3S,3′S), (3R,3′R), and the meso form (3R,3′S). These stereoisomers vary in their occurrence among natural sources; for example, astaxanthin from *Haematococcus pluvialis* is predominantly the (3S,3′S) form, whereas yeast-derived astaxanthin is rich in the (3R,3′R) isomer, and synthetic astaxanthin typically contains all three in approximately a 1:2:1 ratio. In addition to optical isomers, astaxanthin displays geometric (E/Z) isomerism due to multiple conjugated double bonds in its polyene chain, with all-trans (all-E) being the major configuration in nature and 9-cis, 13-cis, and 15-cis isomers reported under certain conditions such as heat or light exposure. The distribution of these isomers can influence physicochemical properties such as solubility, stability, and antioxidant activity, and may therefore affect bioavailability and biological function. The hydroxyl groups on the terminal rings can also form mono- or diester derivatives with fatty acids, which are the predominant forms in many natural sources and further contribute to the diversity of astaxanthin compounds observed in biological systems (Adıgüzel and Ülger, 2024; Nishida et al., 2023; Yu and Liu, 2020). AST has a red color due to the conjugated double bonds in the central region of the molecule. These bonds act as powerful antioxidants, donating electrons to free radicals, which are then converted into other stable compounds, thus preventing the continuation of free radical chain reactions in a wide array of living organisms (Chae et al., 2022). AST exhibits stronger biological activity compared to multiple other antioxidants, due to the fact that it interacts with the cell membrane from both the inner and outer sides (Ambati et al., 2014).

Substantial evidence indicates that AST can downregulate microglial activation and reduce the production of cytotoxic factors. Activated microglia release nitric oxide and other mediators that, when combined with superoxide, form peroxynitrite, a highly reactive species capable of damaging proteins, lipids, and DNA. By inhibiting microglial activation and the secretion of pro-inflammatory cytokines, AST helps preserve neuronal homeostasis. This effect is relevant not only in neurodegenerative pathology but also in aging, where brain inflammation is chronically elevated (Barros et al., 2014). AST is widely acknowledged for its powerful antioxidant properties. Indeed, it has been used as a dietary supplement for several years due to these potent effects. This is particularly significant because the brain, with its high metabolic activity, has a high tendency to oxidative stress. In addition, the brain is rich in oxidizable substrates such as catecholamines and polyunsaturated fatty acids within neuronal membranes, rendering it particularly susceptible to oxidative damage. Over time, oxidative stress is able to impair macromolecules and damage the function of neuron cells. Moreover, oxidative stress is not only a normal aspect of aging but also a contributing factor in various disease conditions. AST is an exceptionally effective antioxidant, with biological activity greater than alpha-tocopherol and beta-carotene (Gbd 2019 Dementia Forecasting Collaborators, 2022).

**FIGURE 1:**
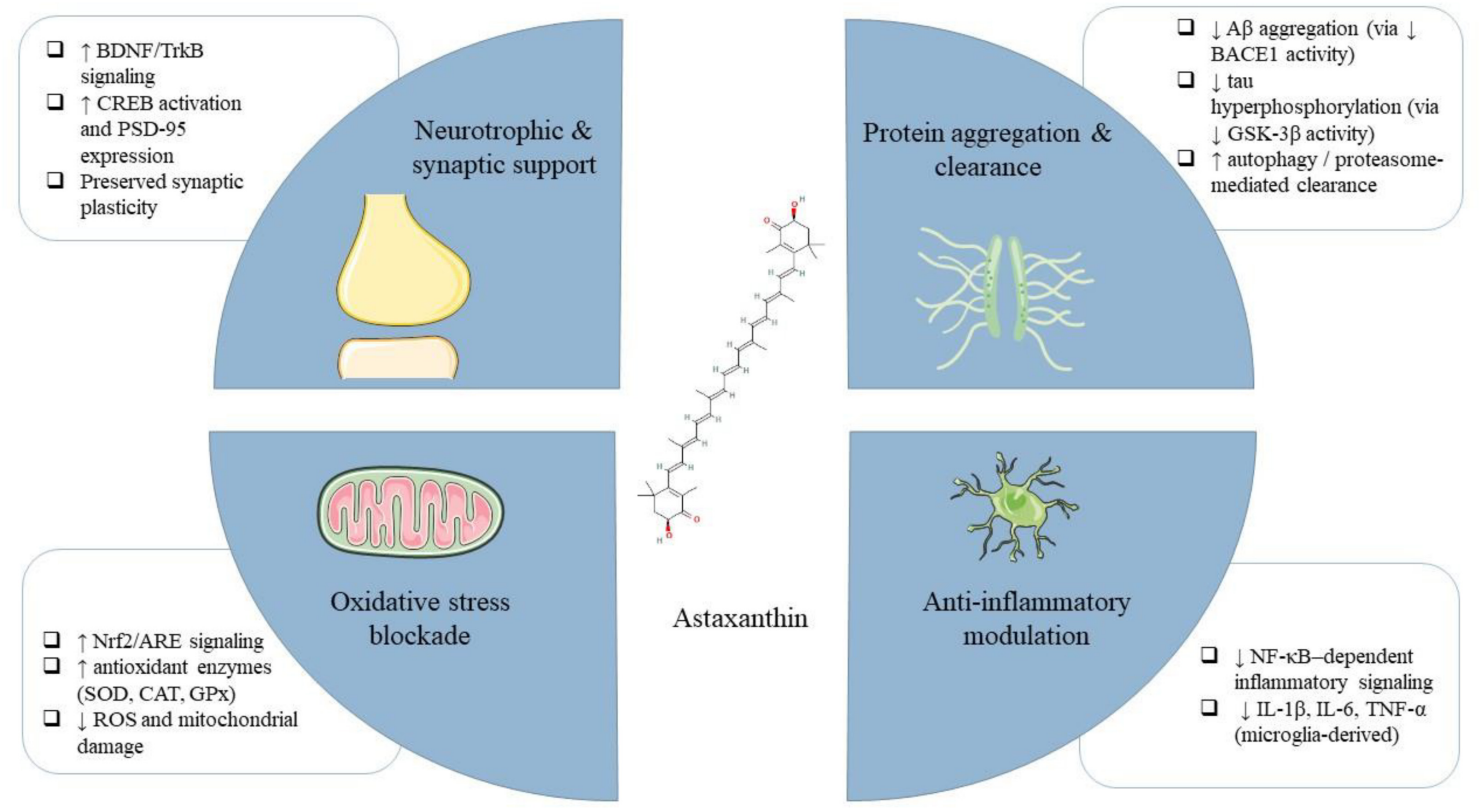
Integrated neuroprotective mechanisms of astaxanthin (AST) in neurodegenerative diseases. AST exerts its effects through multiple pathways: (1) blockade of oxidative stress by reducing reactive oxygen species (ROS), upregulating antioxidant enzymes, and preserving mitochondrial function; (2) modulation of neuroinflammation via inhibition of NF-κB signaling and downregulation of pro-inflammatory cytokines (IL-6, TNF-α, IL-1β); (3) reduction of protein aggregation and enhancement of clearance mechanisms, including inhibition of Aβ and tau accumulation and stimulation of autophagy/proteasome activity; and (4) promotion of neurotrophic and synaptic support by upregulating BDNF/TrkB, CREB, and PSD-95, thereby sustaining neuronal survival and synaptic plasticity.

In prior studies, the antioxidant, anti-inflammatory, and anti-apoptotic properties of AST have been reported to improve the quality of life in diseases ranging from cancer to neurodegenerative diseases. In a diabetic retinopathy model, AST treatment elevated the levels of the antioxidant enzyme heme oxygenase-1 (HO-1) and preserved homeostasis for retinal ganglion cells (Baccouche et al., 2018). Studies have demonstrated that AST can shield keratinocytes from damage caused by ultraviolet exposure by reducing the levels of oxidative markers, such as inducible nitric oxide synthase (iNOS), and pro-inflammatory markers like IL-1β and TNF-α (Yoshihisa et al., 2014). Furthermore, a clinical study revealed that oral AST supplementation provides protection against ultraviolet-induced skin injury (Ito et al., 2018b). Furthermore, AST helps regulate and maintain lipid metabolism homeostasis (Bhuvaneswari et al., 2010). Additionally, AST influences the progression of cancer and cardiovascular diseases through modulating apoptosis-related factors and regulating cell proliferation (Fan et al., 2017). Emerging evidence suggests that AST may offer neuroprotective benefits by addressing key mechanisms implicated in neurodegenerative diseases. Further clinical and/or preclinical studies have demonstrated its potential to decrease oxidative stress, modulate neuroinflammatory pathways, and enhance mitochondrial function—all of which are important to maintaining neuronal health. Additionally, AST has been shown to influence autophagy and amyloid aggregation, processes central to the pathogenesis of diseases like AD and PD. Its neuroprotective effects are further supported by studies showing its ability to improve the function of cognitive and motor systems in animal models of neurodegeneration. These multifaceted mechanisms highlight the potential of AST as a potential agent for protecting against neurodegenerative disorders. Some of the mentioned molecular mechanisms are visually summarized in Figure 1, which illustrates AST’s modulation of key pathways such as Nrf2/ARE, PI3K/Akt, and NF-κB in promoting neuroprotection.

## 6 Therapeutic role of AST in AD

Numerous studies have explored the roles of AST as a therapeutic option in the AD context, highlighting its ability to ameliorate oxidative stress, improve cognitive function, and decrease disease-related pathophysiological markers (Table 1). AST has been shown to influence core pathological mechanisms in neurodegenerative disease (e.g., AD), including the reduction of tau hyperphosphorylation and suppression of BACE1 activity, both of which are key contributors to neurofibrillary tangle formation and Aβ production, respectively (Fanaee-Danesh et al., 2019; Shi and Zhao, 2024). Delivery strategies for improving AST bioavailability and neuroprotective effects presented in Table 2.

**TABLE 1:** The beneficial effects of AST on the biochemical and molecular mechanisms of action and brain function in neurodegenerative diseases.

Name of the disease	Participants	Treatment investigated	Dosage/ duration of treatment	Results	References
AD (AST delivery)	OECs + *in vitro* tests	AST-loaded stealth lipid nanoparticles (AST-SSLNs)	<200 nm nanosystem; parenteral formulation	Improved bioavailability, maintained antioxidant activity, long-term stability, no significant toxicity	Santonocito et al., 2021
AD (oxidative stress, senescence)	ICR mice + PC12 cells + Aβ25–35	AST	*In vivo*: 20 mg/kg/day by oral gavage for 14 days’ pre-treatment + 7 days’ post Aβ injection *In vitro*: ∼10 μM AST (and other tested concentrations) for 48 h exposure	AST improved cognition (shorter escape latency, more platform crossings, more time in target quadrant), prevented hippocampal neuronal loss; ↓ ROS and MDA, ↑ SOD and GSH-px; ↑ Bcl-2/↓ Bax; ↑ SIRT1/PGC-1α; reduced senescence and apoptosis in PC12 cells	Liu et al., 2023
AD (APP/PS1)	APP/PS1 mice	Free AST and AST–DHA	Dietary intake; unspecified duration	↓ Aβ, improved cognition, altered ganglioside metabolism; AST-DHA affected simple GLS more	Wang Z. et al., 2023
AD (APP/PS1)	APP/PS1 mice	AST and AST–DHA (diesters)	2 months	AST-DHA > AST in improving cognition; ↓ tau phosphorylation, oxidative stress, and inflammasome activation	Che et al., 2018
AD (AppNL-G-F)	AppNL-G-F mice	AST	From 5 to 6 weeks old; cognition tested at 6 months	↓ Aβ42, pTau; ↑ PV-positive neurons, microglia; ↑ glutathione; 4-HNE remained elevated	Hongo et al., 2020
AD (AlCl3-induced)	Rats (AlCl3-induced AD)	AST	5, 10, 15 mg/kg/day, oral, 6 weeks	Dose-dependent ↑ cognition; ↓ Aβ1-42, MDA; ↑ Nrf2, miRNA-124, serotonin; ↓ AChE, MAO	Hafez et al., 2021
AD (BBB/Aβ clearance)	pBCECs + 3xTg AD mice	AST + Bexarotene	*In vitro* and *in vivo*	↓ Aβ oligomers; ↑ ADAM10 and sAPPα; enhanced Aβ clearance at BBB	Fanaee-Danesh et al., 2019
AD (Aβ1-42-induced)	Wistar rats	AST powder vs. extract vs. vitamin E	10 mg/kg/day, 30 days oral	AST powder >other forms in ↑ cognition and ↓ oxidative stress	Taksima et al., 2019
AD (scopolamine model)	Adult male mice	AST	Duration unspecified (oral); *in vitro* neuroinflammation test	↓ AChE and inflammation; ↑ memory; restored Akt signaling; enhanced galantamine effects	Babalola et al., 2023
AD (CVD-associated)	Zebrafish	AST, Donepezil, MMP-13 inhibitor	AST 10–20 mg/L, 21 days	↓ Aβ, nitrite, AChE, MMP-13 activity; reduced blood–brain barrier leakage	Paramakrishnan et al., 2023
PD (rotenone model)	SK-N-SH cells	Squid-derived AST	Dose-dependent; duration not stated	↓ ROS, ↑ mitochondrial function; protected against rotenone-induced cytotoxicity	Blesa and Przedborski, 2014
PD (MPTP-induced)	Young + aged mice	AST	–	Protected SNpc neurons in young mice; no effect on TH in aged mice	Anguchamy and Muthuvel, 2023
PD (MPTP-induced)	Male C57BL/6J mice (*n* = 40, 5 groups)	DHA-acylated astaxanthin ester (DHA-AST), non-esterified AST, and DHA + AST combination	Oral gavage, 40 mg/kg AST-equivalent/day for 2 weeks before and during MPTP (30 mg/kg i.p. for 8 days)	DHA-AST most effectively improved motor performance (rotarod), preserved TH-positive dopaminergic neurons, suppressed mitochondrial apoptosis (↓ Bax/Bcl-2 ratio, ↓ Cyt c release, ↓ Caspase-9/3 activation), and inhibited JNK/p38 MAPK signaling. All AST forms reduced oxidative stress, but DHA-AST showed superior anti-apoptotic and neuroprotective effects.	Wang et al., 2020
MS (cuprizone model)	Wistar rats	AST	3 mg/kg/day oral + 0.6% CPZ, 4 weeks	↓ Demyelination, oligodendrocyte loss; ↑ muscle strength; validated by RT-PCR and IHC	Lotfi et al., 2021
MS (EAE model)	Female C57BL/6 mice (*n* = 32, 4 groups)	Astaxanthin pretreatment (3 weeks or 1 week before EAE induction)	0.4% in chow (∼400 mg/kg/day) for 5 weeks	Reduced clinical scores, delayed disease onset, ↓ IFN-γ, IL-6, IL-17, ↑ IL-10, suppressed lymphocyte proliferation, increased Tregs, and reduced CD4+/CD8+ infiltration in brain and spinal cord.	Bidaran et al., 2018

AD, Alzheimer’s disease; PD, Parkinson’s disease; AST, astaxanthin.

**TABLE 2:** Delivery strategies for improving AST bioavailability and neuroprotective effects.

Delivery method	Formulation/example	Advantages	Neuroprotective effects observed	Reference(s)
Liposomes	PEGylated-liposomal AST (<100 nm diameter)	Improved solubility, enhanced brain delivery	Reduced Aβ and formaldehyde accumulation; rescued learning and memory in APP/PS1 mice	Gu et al., 2024
Nanoparticles/ NLCs	AST-loaded nanostructured lipid carriers (NLCs)	Stable intranasal delivery, high brain targeting	Lowered oxidative stress, amyloid pathology, neuroinflammation in AD rats	Shehata et al., 2023
Esterified AST	DSS-acylated AST monoester (AST-DHA)	Better absorption and metabolic stability	Greater cognitive improvement, reduced Aβ and tau pathology via restored autophagy in APP/PS1 mice	Wang et al., 2024
Free AST	Unformulated, non-esterified AST	Naturally occurring, dietary use	Modest systemic presence; limited brain penetration	Mercke Odeberg et al., 2003
Combination therapies	AST + RXR agonist (bexarotene)	Synergistic lipid regulation and Aβ clearance	Enhanced sAPPα production and Aβ transport	Fanaee-Danesh et al., 2019

A study has described the preparation of the novel AST-SSLNs, which a stealth lipid nanoparticles loaded with AST, to improve the bioavailability of AST, revealing a great potential of AST as a potent natural antioxidant and a novel therapy for AD. AST-SSLNs that are prepared by the solvent-diffusion method had the best technological characteristics for parenteral use (particle size < 200 nm). These nanoformulated astaxanthin systems demonstrated acceptable biocompatibility and exerted antioxidant and anti-inflammatory effects in neural cell models, supporting their potential utility for neuroprotective applications. This shields the AST from environmental factors and keeps it protected over time, which was verified by long-term stability and UV stability studies. In addition, the antioxidant activity of AST was preserved by SSLNs as confirmed by oxygen radical absorbance capacity (ORAC) assay (Santonocito et al., 2021; Table 1). Another study proposed that the augmentation of antioxidant capacity could be a good potential target for AD therapy. Liu et al. (2021) investigated the neuroprotective effects of AST on AGE-induced neuronal senescence and apoptosis, which are induced by oxidative stress from Aβ25-35 peptide, in order to prevent cognitive impairment. The ICR mice were used to establish an AD model *in vivo*, while PC12 cells (treated with Aβ25-35) were used to build an *in vitro* AD model. The Morris water maze test was performed for behavioral evaluations in mice, and Nissl staining was performed for assessing morphological changes in hippocampal neurons. The Aβ25-35 model caused cognitive deficits, hippocampal damage, reduced Bcl-2, increased Bax, lower antioxidant enzyme activity, and elevated ROS/MDA. AST treatment reversed these effects. In addition, the treatment of nicotinamide (NAM), a form of vitamin B3, suppressed the activity of sirtuin-1 (SIRT1), a regulator of cellular stress resistance, and peroxisome proliferator-activated receptor gamma coactivator-1 alpha (PGC-1α), a key transcriptional coactivator involved in mitochondrial biogenesis and energy metabolism. This has been related to cognitive impairment and neuronal degeneration of the hippocampus. Exposure to Aβ25-35 *in vitro* resulted in increased oxidative stress, senescence, and apoptosis in PC12 cells; AST mitigated these effects by decreasing oxidative stress and improving cellular conditions. PC12 cells in the Aβ+ NAM group presented the highest degree of senescence and apoptosis following NAM treatment (Liu et al., 2023). A recent study described the impact of docosahexaenoic-acid-acylated AST diesters (AST-DHA) on the AD under the APP/PS1 model. Through LC-MS and molecular biology methodologies, the researchers compared the metabolism of cortical ganglioside (GLS) to free-AST (F-AST) and AST-DHA. AST supplementation in behavioral and immunohistochemical studies dramatically ameliorated cognitive impairment and Aβ deposition. Analysis of 84 GLS molecular species by LC-MS indicated a simultaneous decrease of total GLS levels, including a decrease of complex gangliosides and an increase in their simpler forms (GM3 and GM1a) in these tumors. F-AST significantly influenced complex GLS, whereas AST-DHA mainly adjusted simpler GLS. Notably, it was found that there are significant changes in some specific gangliosides, such as OAc-GQ1a(38:1), OAc-GQ1a(36:1), GD1a(36:1), and GM3(38:1), which were proven to be AD potential biomarkers. In addition, AST dietary intervention upregulated GLS synthesis gene mRNA expression (soat, st3gal5, st3fal2, st8sia1, b3galt4, and siae) while downregulating GLS catabolism gene hexa expression (Wang Z. et al., 2023).

In another study, Che et al. (2018) studied the effectiveness of synthesized docosahexaenoic-acid-acylated AST diesters (ASTDHA) in slowing the progression of AD. AST and AST-DHA have been given for 2 months to APP/PSEN1 (APP/PS1) double-transgenic mice. The radial 8-arm maze and Morris water maze tests indicated AST-DHA had significantly better performance than AST in increasing cognitive abilities, including learning and memory in the APP/PS1 mice. A mechanistic evaluation also revealed that AST-DHA better than AST ameliorated all parameters of oxidative stress and tau hyperphosphorylation and inhibited neuroinflammatory responses compared with AST+APP/PS1 by regulating the expression and activity of inflammasomes. It is also observed that when AST is given to AppNL-G-F/NL-G-F mice starting at around 5–6 weeks of age, it significantly enhances memory performance. Moreover, AST supplementation reduced Aβ42 deposition and the area fraction of pTau-positive cells, while increasing the density of PV-positive neurons and microglial response to Aβ42 deposition in the hippocampus. While AST elevated total glutathione (GSH) levels, markers of oxidative stress, such as 4-hydroxy-2,3-trans-nonenal (4-HNE) protein adducts, remained elevated in treated mice (Hongo et al., 2020).

Hafez et al. (2021) evaluated the effects of different doses of AST on the cerebral cortex and hippocampus of AD rat models. A total of 75 mg/kg AlCl3 was given orally every day to induce cognitive dysfunction in an AD-like rat model for 6 weeks. Subsequently, the rats were administered DMSO (control) or the different doses of AST (e.g., 5, 10, and 15 mg/kg) over the next 6 weeks. AST significantly improved Morris water maze test performance in a dose-dependent manner, inhibited the accumulation of amyloid β1-42 and malondialdehyde levels. Moreover, treatment with AST reduced activities of acetylcholinesterase and monoamine oxidase, as well as β-site APP cleaving enzyme 1 (BACE1) expression. In addition, the expression of miRNA-124, acetylcholine, nuclear factor erythroid-2-related factor 2 (Nrf2), and serotonin was significantly increased by AST treatment. Another study aimed to assess the effects of a retinoid X receptor agonist, bexarotene (Bex), and AST (peroxisome proliferator-activated receptor α agonist and antioxidant) on cellular cholesterin metabolism, amyloid precursor protein (APP) processing, and Aβ production, as well as their transport over the blood-brain barrier. This work utilized an *in vitro* model with primary porcine brain capillary endothelial cells (pBCEC) and 3xTg AD mice. The findings indicated that AST/Bex treatment restrained transcription and activity of the amyloidogenic enzyme BACE1, consequently decreasing Aβ oligomers as well as 80-kDa intracellular APP/Aβ species. AST/Bex also upregulated the non-amyloidogenic enzyme ADAM10 and enhanced the secretion of soluble (s)APPα in pBCEC. Furthermore, AST/Bex enhanced Aβ clearance to the apical/plasma side in the *in vitro* model of the blood-brain barrier. AST/Bex also upregulated the expression of ABCA1, LRP1, and/or APOA-I, thereby facilitating cholesterol efflux partially through PPARα/RXR activation, and downregulated cholesterol biosynthesis and esterification. LRP1 silencing or ABCA1 inhibition by probucol reversed the effects of both AST and Bex on APP/Aβ species levels in pBCEC. Interestingly, Bex treatment induced APOE and ABCA1 expression in murine brain capillary endothelial cells (mBCEC) in 3xTg AD mice. Transformingly, the original figure was transformed to be compared with the found results, demonstrated by total BACE1 expression of mBCEC from AST/Bex-treated 3xTg AD mice, that are lower than the BACE1 expression level in 3xTg AD mice and non-transgenic. Moreover, AST/Bex treatment decreased oligomers of Aβ in mBCEC and Aβ species in both soluble and insoluble brain parts of 3xTg AD mice (Fanaee-Danesh et al., 2019).

Taksima et al. (2019) also performed a study on the activity of AST obtained from shrimp shells (*Litopenaeus vannamei*) and AST powder on Wistar rats with AD. In Aβ1–42–induced rodent models of Alzheimer’s disease, astaxanthin treatment—administered in different formulations, consistently improved cognitive performance and reduced oxidative stress, neuroinflammation, and histopathological hallmarks compared with untreated or vehicle-treated controls. The treatments have been given for 30 days and were orally administered. On days 14 and 29 after injection, behavioral assessments were conducted, with animals euthanized on day 31 to harvest both the hippocampus and cortex for further analyses. Results have indicated that the group induced with Aβ1–42 exhibited severe cognitive deficiency and memory impairment, verified by the Morris water maze task, novel object recognition task, and novel object placement task. They also had increased oxidative stress, demonstrated by higher levels of glutathione peroxidase (GPx), lipid peroxidation products (MDA), protein oxidation, and superoxide anions in both cortex and hippocampus in this group. The AST powder (10 mg/kg body weight) greatly enhanced cognitive function and memory, whereas oxidative stress declined significantly, greater than either AST extract or vitamin E at equal dosages. In another study, the mechanisms underlying the neuroprotective effects of AST were investigated in the scopolamine-induced AD model of mice. Following the treatment period (14 days), short-term memory, hippocampal tissue integrity, oxidative stress scores, and inflammatory markers were evaluated. The findings showed that AST considerably improved short-term memory, lowered brain acetylcholinesterase activity, and had neuroprotection and anti-amyloidogenic effects. Moreover, AST significantly reduces the pro-inflammatory factors and oxidative stress, as well as restores the Akt-1 and phosphorylated Akt signaling pathways responsible for the control of pathological aberrant tau proteins. AST also increases the anti-inflammatory and antioxidant action of galantamine (Babalola et al., 2023). An additional investigation examined the impact of AST on cerebrovascular disease (CVD)-associated AD using a zebrafish model, with a specific focus on the inhibition of matrix metalloproteinase-13 (MMP-13) activity. The induction of CVD was achieved by administration of streptozotocin (STZ) into the peritoneum and cerebrum. Over a period of 21 days, the zebrafish received treatment with AST at concentrations of 10 and 20 mg/L, along with donepezil at 1 mg/L and the MMP-13 inhibitor CL-82198 at 10 μM. Cognitive function was assessed through various tests, including light and dark chamber assessments, a color recognition task, and a T-maze test. Furthermore, biomarkers related to AD were evaluated by measuring cerebral leakage of Evans blue, concentrations of tissue nitrite, aggregation of αβ, function of MMP-13, and activity of acetylcholinesterase. Findings demonstrated that treatment with AST led to significant enhancements in both behavioral and biochemical parameters (Paramakrishnan et al., 2023). More recently, astaxanthin has been evaluated in a scopolamine-induced rat model of Alzheimer’s disease with a focus on both behavioral and molecular outcomes. In this study, AST administration significantly improved learning and memory performance while attenuating hippocampal oxidative damage and inflammatory responses. These effects were associated with activation of the Nrf2 antioxidant pathway and suppression of NF-κB signaling. Notably, pharmacological blockade of opioid and benzodiazepine receptors partially reversed AST-mediated neuroprotection, suggesting that receptor-dependent mechanisms may contribute to its cognitive benefits beyond classical antioxidant activity (Rastinpour et al., 2025).

Overall, AST demonstrates considerable potential as a neuroprotective agent in AD. Both *in vivo* and *in vitro* studies show AST’s ability to enhance cognitive function, reduce neuroinflammation, and modulate key neurodegenerative pathways. These promising results suggest AST could serve as an effective adjunct in AD therapy, though further clinical studies are needed to establish its long-term benefits. Across AD models, AST demonstrates consistent antioxidant and anti-inflammatory benefits; however, the outcomes vary depending on formulation (free AST vs. DHA-conjugates vs. nanoparticle systems) and dosage. While lipid- and DHA-conjugated forms tend to outperform free AST in cognitive improvement and plaque reduction, no standardized regimen exists, making comparisons across studies difficult. Importantly, clinical evidence in humans remains extremely limited, meaning that promising animal results should be interpreted cautiously until validated in well-controlled trials.

**FIGURE 2:**
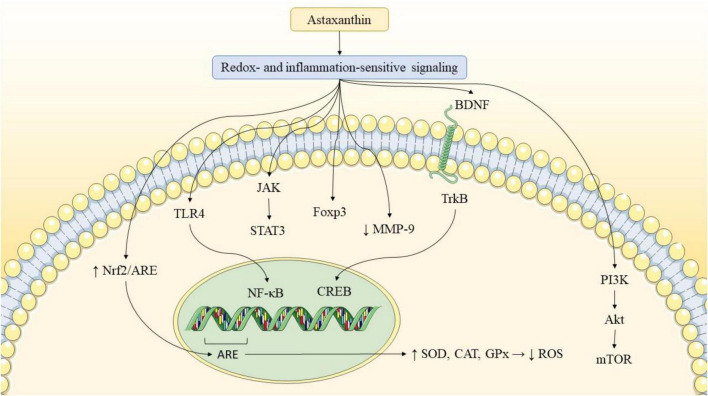
AST’s modulation of neurodegenerative pathways. AST reduces reactive oxygen species (ROS) and upregulates antioxidant enzymes (GPx, SOD, CAT). It suppresses pro-inflammatory mediators (NF-κB, IL-6, TNF-α, IL-1β), inhibits Aβ aggregation, promotes Aβ clearance, and reduces tau phosphorylation. AST also enhances synaptic function by upregulating BDNF, CREB, and PSD-95, contributing to improved cognitive and neuronal outcomes. AST exerts indirect neuroprotective effects primarily through redox- and inflammation-sensitive signaling pathways (e.g., Nrf2/ARE activation and NF-κB inhibition), leading to downstream antioxidant, anti-inflammatory, and synaptic-supportive outcomes.

Figure 2 provides an integrated overview of AST’s effects on pathological processes relevant to neurodegenerative diseases, including oxidative stress, neuroinflammation, and synaptic dysfunction, which are particularly relevant in AD and PD pathology. While Figures 1, 2 summarize AST’s modulation of oxidative and inflammatory pathways, it is important to note that these models are derived largely from preclinical studies. Differences in dosage, formulation, and disease models may limit direct translation to clinical settings, underscoring the need for careful interpretation.

## 7 Therapeutic role of AST in PD

Astaxanthin has been studied as a possible treatment strategy for PD mainly because of its ability to oppose oxidative stress, decrease neuroinflammation, and improve mitochondrial function. Emerging evidence suggests that AST may reduce α-synuclein aggregation and its associated cytotoxicity (Nguyen et al., 2025). By modulating oxidative stress and mitochondrial integrity, AST indirectly influences α-synuclein clearance mechanisms, potentially limiting the formation of Lewy bodies (Paramakrishnan et al., 2023).

Anguchamy and Muthuvel (2023) performed the experimental PD *in vitro* model, and evaluated the protective effects of AST treatment on SK-N-SH human neuroblastoma cell lines, via exposure to rotenone as a toxic agent. Results emphasized the strong antioxidant potential of AST from squid, with a more pronounced ability to neutralize DPPH radical species. Moreover, treatment with AST mitigated cytotoxicity, dysfunction of mitochondria, and oxidative stress induced by rotenone in a dose-dependent manner in SK-N-SH cells. Furthermore, studies have focused on the age-dependent neuroprotective effects of AST. One study examined the impact of AST on neurotoxicity in a PD mouse model of young and aged mice. The results from this study indicated that astrocyte-targeting strategy (AST) clearly protected neurons from MPTP neurotoxin in both the young and aged groups, while the effect of AST was less pronounced in older mice, since it could not prevent the decrease in tyrosine hydroxylase (TH) in the nigrostriatal pathway (Grimmig et al., 2018). In a subsequent study, researchers investigated the effects of DHA-AST, AST, and DHA + AST on PD progression in mice induced by 1-methyl-4-phenyl-1,2,3,6-tetrahydropyridine (MPTP). The rotarod test results revealed that DHA-AST significantly slowed the progression of PD in MPTP-treated mice, outperforming both AST alone and the DHA + AST combination. Further mechanistic analysis showed that all three treatments were effective in reducing oxidative stress in the brain (Wang et al., 2020). Recent investigations using paraquat-induced models of PD have demonstrated AST’s robust protective capacity in both cellular and animal settings. *In vitro*, AST significantly improved the viability of SH-SY5Y cells exposed to paraquat by dampening oxidative stress and curbing apoptosis. *In vivo*, treatment with AST alleviated motor impairments and mitigated the loss of dopaminergic neurons in the substantia nigra pars compacta (SNpc) of C57BL/6J mice. These neuroprotective effects were linked to suppression of paraquat-induced activation of the MAPK signaling cascade, a pathway known to mediate oxidative and apoptotic damage in dopaminergic systems. These results highlight the potential of AST to interrupt molecular triggers of neuronal degeneration in environmentally induced PD models (Wang L. et al., 2023). Another investigation has uncovered a novel mechanism by which AST exerts its protective effects against PD-related neuronal injury. In SH-SY5Y cells challenged with MPP+, AST reversed ER stress-induced apoptosis and restored cell viability. Mechanistically, AST modulated the miR-7/SNCA axis—where miR-7 negatively regulates alpha-synuclein expression. Inhibition of miR-7 or overexpression of SNCA abolished the protective effects of AST, confirming this regulatory pathway. Furthermore, *in vivo* experiments using MPTP-treated mice revealed that AST reversed behavioral deficits, preserved tyrosine hydroxylase (TH) expression, and reduced neuronal apoptosis, even in the presence of miR-7 knockdown. These findings suggest that AST protects dopaminergic neurons through suppression of ER stress and stabilization of the miR-7/SNCA regulatory loop, presenting a promising target for PD therapy (Shen et al., 2021).

In summary, AST demonstrates promising therapeutic potential in PD by targeting key pathological mechanisms such as oxidative stress, neuroinflammation, and mitochondrial dysfunction. Studies on various PD models, including *in vitro* and *in vivo* experiments, highlight AST’s protective effects on dopaminergic neurons against toxins like rotenone and MPTP. Despite its efficacy, particularly in younger animals, AST’s neuroprotective effects appear to diminish with age, highlighting the need for further research to optimize its therapeutic potential. These findings suggest that AST could play a vital role in managing PD, but more clinical investigations are needed to fully establish its benefits, especially for aging populations. Therapeutic outcomes in PD models also highlight age dependency and formulation differences: younger animals consistently show greater neuroprotection, while older models exhibit attenuated responses. Esterified or DHA-bound AST appears to enhance bioavailability and efficacy compared with unmodified AST. Despite encouraging preclinical findings, clinical data are currently absent, and prioritization of early-phase human trials is necessary before any definitive conclusions can be drawn.

## 8 Therapeutic role of AST in MS

Multiple sclerosis is a neurodegenerative disorder where the body’s immune system works against the myelin sheath in the central nervous system, causing nerve damage and disrupting signal transmission. This leads to symptoms such as muscle weakness, coordination problems, cognitive decline, and sensory disturbances. As the disease progresses, neuronal injury increases, contributing to disability. MS shares features with other neurodegenerative diseases, particularly in terms of neuroinflammation and progressive neural degeneration. As previously described, common molecular contributors to neurodegeneration such as oxidative stress, mitochondrial dysfunction, and glial activation also play central roles in MS pathogenesis.

Several investigations have examined the potential role of AST in alleviating key pathological mechanisms of MS. In MS models, AST has demonstrated protective effects against demyelination by preserving oligodendrocyte viability and reducing inflammatory cytokine expression, both of which are critical in maintaining myelin sheath integrity (Lotfi et al., 2021).

Lotfi et al. (2021) also explored the action of AST in protection against demyelinating and oligodendrocytic injury in rats with MS. They used Forty Wistar rats and randomly divided into four separate groups, namely control, administered a standard diet; cuprizone (CPZ) group, administered 0.6% CPZ for 4 weeks, sham group, administered 0.6% CPZ and dimethyl sulfoxide for the same period, and AST group, administered cuprizone CPZ daily along with administered AST at a dose of 3 mg/kg administered 12 h later for 4 weeks. Strength was measured weekly with the behavioral basket test. Luxol Fast Blue (LFB) staining was used to assess myelinating and demyelinating, and myelin density was quantified using ImageJ software. Immunohistochemistry (IHC) methods were also used to assess expression of A2B5 (a marker of oligodendrocyte precursor cells) and MOG. Myelin basic protein (MBP), MOG, and PDGFR-α expression levels were analyzed by RT-PCR. The results indicated that AST treatment alleviated oligodendrocyte damage and disrupted myelin sheaths in the MS model. The AST-treated group showed significant improvement in muscle strength compared to the CPZ and sham groups. Furthermore, RT-PCR and IHC findings confirmed that AST effectively reduced demyelination and oligodendrocyte loss in MS (Lotfi et al., 2021). Furthermore, Bidaran et al. (2018) examined the therapeutic and prophylactic roles of AST in experimental autoimmune encephalomyelitis (EAE), a well-accepted model for studying MS 43. In the chronic EAE model, MOG was utilized to induce disease in female C57BL/6 mice. In the studies of splenocytes, both pro-inflammatory cytokines were significantly reduced after AST treatment along with inhibition of cell proliferation and an increase in FoxP3+ regulatory T cells (Tregs). Generalizing to spinal cord and brain tissues, immunohistochemical analysis showed that infiltrating inflammatory cells were largely restricted to the central nervous system. In addition, AST treatment reduced clinical scores and ameliorated disease severity, further highlighting the protective potential of AST in the MS model. In MS models, AST supplementation consistently reduces demyelination and inflammatory cytokine release, though the doses and treatment durations vary considerably across studies. Compared to AD and PD research, investigations in MS remain fewer in number and are exclusively preclinical. Whether these effects translate to meaningful clinical outcomes, particularly in relapsing–remitting versus progressive forms of MS, remains unknown. Taken together, these therapeutic studies suggest that AST exerts beneficial effects across multiple neurodegenerative conditions. Yet, variability in formulations, dosing strategies, and experimental models complicates direct comparison. Moreover, the hierarchy of evidence remains skewed toward preclinical animal and cell studies, while clinical evidence is sparse and often exploratory. A structured summary of clinical studies is therefore necessary to contextualize these findings. While preclinical evidence is extensive, clinical studies remain scarce and heterogeneous. To date, only a limited number of trials have evaluated the effects of AST in cognitive impairment and neurodegenerative populations. These are summarized in Table 3, which highlights differences in study design, dosing regimens, and outcome measures.

**TABLE 3:** Clinical trials of AST in cognitive function / dementia.

Year	Sample population (age, condition)	AST dose and duration	Outcomes measured	Main findings	Notes / adverse events	References
2012	96 subjects, 45–64 yrs, cognitively normal / mild impairment	6 or 12 mg/day for 12 weeks	Memory (CogHealth battery, GMLT)	Memory improved vs. baseline but not statistically significant	No serious adverse events reported; small effect size	Katagiri et al., 2012
2018	54 subjects, 45–64 yrs, healthy or mild impairment	8 mg/day for 8 weeks	Verbal fluency, Stroop, memory tasks	Improvements in medium-term memory and verbal fluency; age subgroup (>55) less consistent	Tolerated well; no adverse events noted	Hayashi et al., 2018
2018	21 subjects, 30–60 yrs, healthy/mild impairment	6 mg/day AST + sesamin derivative for 12 weeks	Processing speed, psychomotor speed (ADAS-Cog, CNSVS)	Improvement in processing and psychomotor speed vs. controls	Small sample, short duration; no safety concerns noted	Ito et al., 2018a
Completed at 2024	Alzheimer’s disease patients, age 60–90, mild AD under cholinesterase inhibitors	AST vs. placebo for 1 year	MMSE, CASI, clinical dementia rating, NPI etc.	Results pending	Monitoring safety and tolerability included in design	Batool et al., 2022

Beyond classical neurodegenerative disease models, astaxanthin has also demonstrated neuroprotective effects in experimental spinal cord injury, a condition characterized by acute oxidative stress, neuroinflammation, and neuronal loss. In rodent SCI models, AST treatment reduced lipid peroxidation, suppressed pro-inflammatory cytokine release, preserved neuronal and glial viability, and improved functional recovery. These findings further support AST’s capacity to protect neural tissue under severe inflammatory and oxidative conditions. However, because spinal cord injury represents an acute traumatic insult rather than a chronic, progressive neurodegenerative process, such studies were not discussed in detail in the present review, which focuses primarily on neurodegenerative aging and disease. Despite promising preclinical findings, clinical evidence supporting astaxanthin’s neuroprotective effects remains limited. Available human studies are characterized by relatively small sample sizes, heterogeneity in dosing regimens, and short intervention durations, which restrict the ability to draw firm conclusions regarding efficacy and optimal therapeutic windows. Consequently, the translational relevance of current clinical data should be interpreted with caution, and larger, well-controlled trials with standardized dosing strategies are needed.

## 9 Limitations of preclinical evidence

Despite the growing body of preclinical evidence supporting the neuroprotective potential of astaxanthin, several important limitations should be acknowledged. First, the majority of available data are derived from *in vitro* and animal models, which do not fully capture the complexity, heterogeneity, and long-term progression of human neurodegenerative diseases. As a result, the translational relevance of these findings remains inherently limited. In addition, the existing clinical evidence is sparse. Human studies conducted to date generally involve small sample sizes, heterogeneous dosing regimens, and relatively short intervention periods, restricting the ability to draw robust conclusions regarding efficacy, optimal dosage, and treatment duration. Consequently, current clinical findings should be interpreted with caution. Potential publication bias must also be considered, as studies reporting positive effects of astaxanthin are more likely to be published than neutral or negative findings. Furthermore, data on the long-term safety and tolerability of astaxanthin, particularly at higher doses or with prolonged administration, remain limited. Addressing these gaps through well-designed, large-scale clinical trials with standardized protocols and extended follow-up periods will be essential to clarify the therapeutic relevance of astaxanthin in neurodegenerative disorders.

## 10 Future directions

While preclinical evidence strongly supports AST’s neuroprotective properties, translation into clinical application requires well-defined priorities. Future research should focus on rigorously designed randomized controlled trials (RCTs), particularly in early disease stages such as mild cognitive impairment and prodromal Alzheimer’s disease, where neuronal function may still be preserved. These studies should incorporate sensitive biomarkers, including neuroimaging (MRI-based volumetry and functional connectivity), cerebrospinal fluid Aβ/tau ratios, and emerging blood-based markers of oxidative stress and inflammation. Comparable trials in newly diagnosed Parkinson’s disease or relapsing–remitting multiple sclerosis could also clarify AST’s disease-modifying potential. Optimizing delivery strategies remains another priority, given AST’s limited oral bioavailability. Novel formulations such as lipid nanoparticles, esterified derivatives, or intranasal carriers should be systematically compared for pharmacokinetics, blood–brain barrier penetration, and clinical efficacy. Longitudinal studies are also warranted to assess the safety of chronic supplementation, with careful attention to metabolic parameters, liver function, and potential drug–nutrient interactions. Although AST is generally regarded as safe and is available as a dietary supplement, the long-term effects of high-dose or chronic administration remain insufficiently studied. Reported adverse effects in human supplementation trials are rare but may include gastrointestinal discomfort, skin pigmentation, and hormonal modulation at higher doses (Fassett and Coombes, 2011; Yoshida et al., 2010). Furthermore, because AST is a carotenoid with lipophilic properties, there is concern about its potential to accumulate in tissues with prolonged use. Interactions with lipid metabolism and fat-soluble vitamins also warrant systematic investigation. To date, most clinical studies have been of short duration (4–12 weeks), and long-term safety data in neurodegenerative populations are lacking (Choi et al., 2011; Satoh et al., 2009). Rigorous monitoring of metabolic, hepatic, and endocrine parameters will be essential in future trials to ensure safety alongside efficacy. Finally, combining AST with lifestyle interventions (e.g., exercise, dietary modification) or complementary nutraceuticals may enhance its therapeutic impact. Such multimodal approaches align with the multifactorial nature of neurodegenerative disorders. By prioritizing targeted clinical trials, biomarker-based endpoints, and long-term safety monitoring, future research can bridge the current translational gap and clarify AST’s place in neuroprotective therapy.

## 11 Conclusion

Astaxanthin has emerged as a uniquely versatile carotenoid with potent antioxidant, anti-inflammatory, and mitochondrial-supporting properties that distinguish it from many conventional antioxidants. Unlike compounds such as vitamin E or beta-carotene, AST readily crosses the blood–brain barrier and acts across both hydrophilic and lipophilic environments, enabling broad neuroprotective effects. Evidence from preclinical studies consistently supports its role in reducing oxidative stress, suppressing neuroinflammation, and preserving neuronal function in models of Alzheimer’s disease, Parkinson’s disease, and multiple sclerosis. What sets this review apart is its focus on the translational dimension: highlighting recent advances in delivery systems to overcome bioavailability challenges and identifying gaps that must be addressed through clinical research. The therapeutic promise of AST lies not only in its molecular potency but also in its potential integration into multi-target interventions for complex neurodegenerative disorders. To realize this potential, the field must prioritize well-structured clinical trials with biomarker-based outcomes and long-term safety evaluation. By bridging mechanistic insights with clinical relevance, AST stands out as a promising adjunct in the pursuit of effective neuroprotective strategies.
